# Biodiversity and culture of prokaryotes inhabiting haloalkaline and meromictic Soap Lake, Washington, USA

**DOI:** 10.3389/fmicb.2025.1620605

**Published:** 2025-08-06

**Authors:** Olivia J. M. Vanderlaan, Emily M. Simmons, Kelli M. Damman, Makenna D. Waddell, Savannah F. Ross, Amanda D. Armstrong, Mackenzie L. Walker, S. Josiah Sattley, W. Matthew Sattley

**Affiliations:** ^1^Department of Biology, Indiana Wesleyan University, Marion, IN, United States; ^2^The King’s Academy, Jonesboro, IN, United States

**Keywords:** Soap Lake, meromictic lake, soda lake, hypersaline, halophilic bacteria, alkaliphilic bacteria, haloalkaliphile

## Abstract

Despite their potential for harboring novel microorganisms exhibiting beneficial metabolisms or that produce useful products for biotechnology and industry, alkaline lakes and soils are among the least studied extreme environments. With its high productivity and meromictic water column, haloalkaline Soap Lake (Washington, USA) is among the most intriguing soda lakes in the world. We sampled the water column of Soap Lake and used both culture-based and culture-independent (16S rRNA amplicon-based) methods to analyze the microbial diversity of both its oxic and anoxic waters. Cultivable aerobic heterotrophs were specifically targeted in enrichment cultures, and over 100 isolates were obtained. Small-subunit rRNA gene sequences were obtained for isolates that exhibited diverse colony morphologies and grew well on alkaline media containing varying concentrations of NaCl, and two of these isolates were chosen for in-depth characterization: strain 12SL-E129, which aligned within the genus *Roseinatronobacter*; and strain SL14, of the genus *Vibrio*. Both strains grew optimally at or above pH 9 and were halophilic—no growth was evident in the absence of NaCl for either isolate. In addition, strain SL14 exhibited impressive cold adaptation, showing a faster growth rate at 0°C than at 37°C. Community (16S rRNA) analyses conducted on Soap Lake water samples from both the mixolimnion (3 m) and the monimolimnion (23 m) revealed an extensive diversity of *Bacteria*, with the shallower depth dominated by species of *Pseudomonadota* (especially *Alphaproteobacteria*), *Actinomycetota*, and *Bacteroidota*; Deep anoxic waters were dominated by *Bacillota*, including many taxa containing endospore formers, as well as a marked increase in sulfate-reducing *Deltaproteobacteria*. Only low numbers of *Archaea* were identified in both the upper and lower waters of Soap Lake. Our data suggest that despite its extreme conditions (high alkalinity, steep salinity gradient, and reportedly extraordinarily high sulfide concentrations in the monimolimnion), Soap Lake is a highly productive aquatic system supporting thriving and diverse bacterial communities.

## 1 Introduction

Chemoorganotrophic bacteria that inhabit environments of high salinity and alkalinity are of considerable economic and environmental interest due to their production of useful haloalkaline-active enzymes and ability to catabolize organic compounds under the extreme physicochemical conditions typical of many commercial industrial processes (Pollock et al., 2007; Sarethy et al., 2011; Mamo and Mattiasson, 2020; Rawat et al., 2024; Sarwa et al., 2024; Kour et al., 2025). The accumulation of organic-rich, alkaline wastewaters of elevated salinity is a repercussion of manufacturing practices routinely employed by the agricultural, bio- and petrodiesel, and textile industries (Paul et al., 2014). With this in mind, there has been increasing interest in recent decades in investigating natural haloalkaline ecosystems for novel microorganisms whose metabolisms facilitate carbon mineralization and nutrient cycling at high salinity and pH.

**Figure 1:**
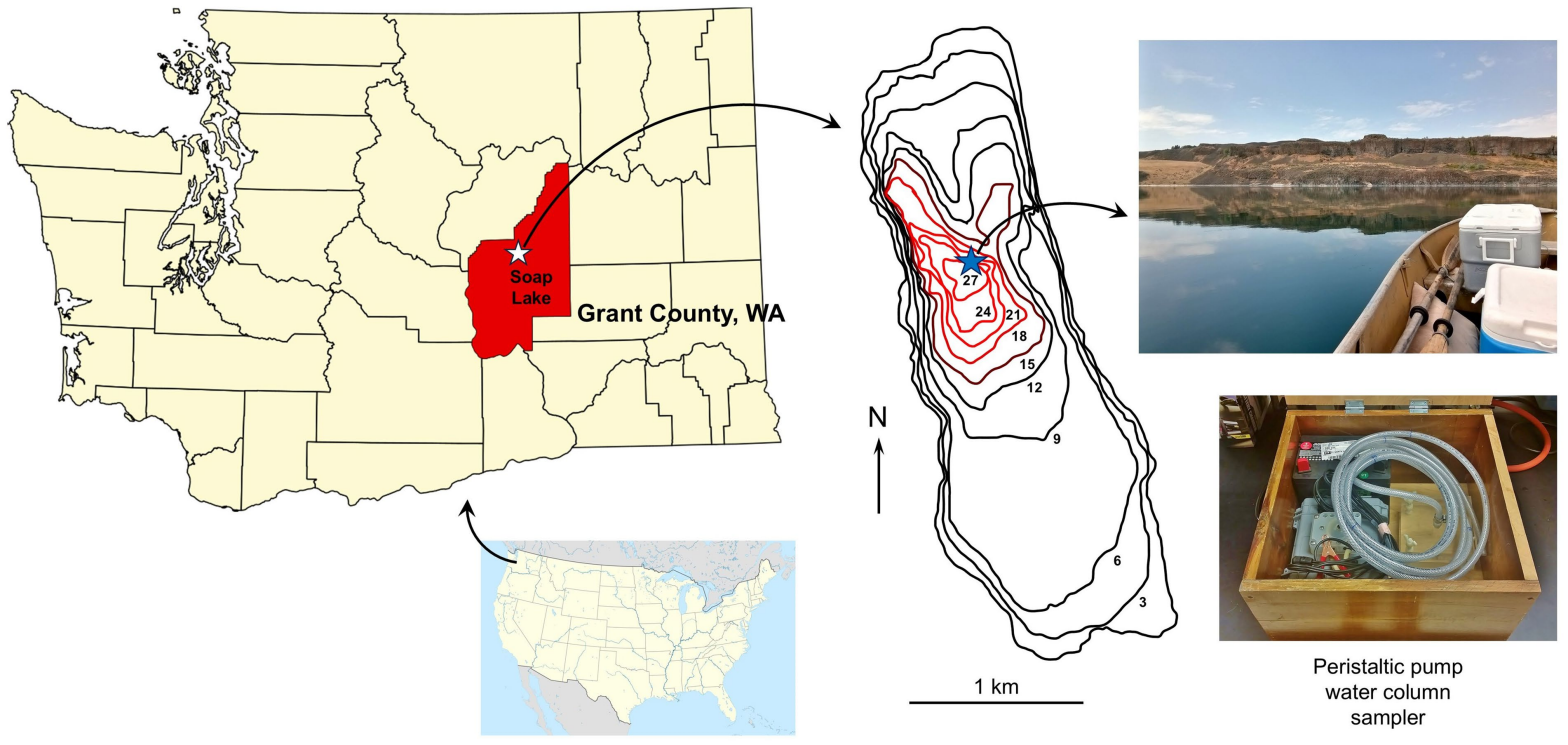
Geography and sampling of Soap Lake, east central Washington, USA. Soap Lake is the final and lowest elevation lake in a chain of eight lakes in the Lower Grand Coulee Basin. The lake has a closed basin and no outlet. The bathymetric map [adapted from Edmondson and Anderson (1965)] indicates lake depths (m), with the anoxic monimolimnion indicated with red lines. The blue star indicates the sampling site. The water column was sampled on a calm day using a fine-resolution, peristaltic pump-driven sampling device (Sattley et al., 2017).

Soap Lake, located in east central Washington state (USA, Figure 1), is the southernmost of a chain of eight closed basin lakes formed from a glacial outlet channel (Edmondson and Anderson, 1965). The lakes drop progressively in elevation, with Soap Lake being the lowest, and it is one of just a few haloalkaline, meromictic, soda lakes in North America (Kallis et al., 2010). In addition to its alkaline waters that approach pH 10 (Mormile, 2014), the water column of Soap Lake is chemically stratified. The mixolimnion, which is comprised of surface waters down to about 19 m, is relatively warm (11–21°C), brackish, and well oxygenated, whereas the monimolimnion (21–27 m) is colder (6–10°C), anoxic, hypersaline, and contains unprecedentedly high levels of dissolved sulfide that have been reported to exceed 150 mM (Sorokin et al., 2007). Between these layers, the chemocline contains both dissolved O_2_ and sulfide at contrasting steep gradients, a condition in meromictic lakes that supports the proliferation of sulfur-oxidizing chemolithotrophic bacteria (Sattley and Madigan, 2006; Sorokin et al., 2007; Zadereev et al., 2017).

Although the pH of Soap Lake is stable at 9.8 throughout the water column, the salinity increases with depth from 14 g L^−1^ at the surface to 20 g L^−1^ at the chemocline, followed by a sharp increase to 140 g L^−1^ in the monimolimnion (Sorokin et al., 2007). In addition, dissolved solids increase dramatically from the chemocline to the bottom of the lake (Sorokin et al., 2007), and because of this, the deep waters are more turbid and dark yellow in color and are accompanied by a strong odor of sulfide and decomposing organic matter. The alkalinity of Soap Lake is a consequence of high levels of dissolved carbonate and sulfate, and it is the latter that supports the high rate of biological sulfate reduction responsible for the extraordinary sulfide concentrations in the anoxic brine (Dimitriu et al., 2008).

Despite high rates of biological productivity (Asao et al., 2007, 2011) and a unique physicochemistry that suggest the presence of a diversity of potentially novel, polyextremophilic microbes, microbiological investigations of Soap Lake have been limited. The few culture-based studies of Soap Lake conducted thus far have focused primarily on anoxygenic phototrophic bacteria (Asao et al., 2007, 2011) and chemolithotrophic sulfur-oxidizing bacteria (Sorokin et al., 2007), as well as studies of isolates having potential for bioenergy (Mormile, 2014; Paul et al., 2014) or that play a role in metal reduction (Pollock et al., 2007; VanEngelen et al., 2008). Therefore, a broader survey of the cultivable chemoorganotrophic bacteria inhabiting Soap Lake is warranted and may yield isolates that, for example, produce useful alkaline-active enzymes or employ metabolisms beneficial for bioremediation purposes.

To investigate the microbial communities inhabiting Soap Lake and potentially unveil microorganisms having unique lineages or useful properties, we sampled the Soap Lake water column and have employed a combination of culture-based and culture-independent analyses to examine waters from both its mixolimnion and monimolimnion. In a previous metagenomic analysis of Soap Lake water, Hawley and Hess (2014) reported “relatively low” microbial diversity. However, this report was based on only a single surface water grab sample, and other studies have indicated an increase in biodiversity with depth in Soap Lake (Sorokin et al., 2007; Dimitriu et al., 2008). In the present investigation, we report a highly heterogeneous picture of Soap Lake microbial communities characterized by distinct assemblages of taxa in mixolimnion (3 m) waters versus monimolimnion (23 m) waters. In addition, we have isolated numerous strains of aerobic chemoorganotrophic bacteria from throughout the Soap Lake water column and herein provide morphological, physiological, and phylogenetic characterization of two novel isolates as part of an ongoing effort to better understand the microbiology of this unique ecosystem.

## 2 Materials and methods

### 2.1 Sample collection and strain isolation

Soap Lake water column samples from the surface to 24 m were collected in 1-m intervals at 47°24.39′N, 119°29.57′W on 28 July 2021 using a peristaltic pump-driven limnological sampling device (Figure 1), as previously described (Sattley and Madigan, 2010; Sattley et al., 2017). The wind during sampling was calm (Figure 1) and, thus, sampling drift was minimal. The parafilm-sealed, completely filled, Nalgene® bottle samples were kept cold but unfrozen during transport to the laboratory, whereupon they were stored at 4°C. Primary enrichment cultures for aerobic chemoorganotrophs were established by transferring 1 mL aliquots from each of the 25 lake water samples to 125-ml Erlenmeyer flasks containing 50 mL of sterile modified R2A medium (Reasoner and Geldreich, 1985). The modified R2A medium contained no soluble starch and was adjusted to pH 9.5 (buffered with 10 mM CAPSO) and amended with either 0, 1, 1.5, or 2% (w/v) NaCl.

After 1–3 weeks of incubation at 10°C, liquid cultures established from all depths of lake water were turbid. The cultures were systematically streaked for isolation on agar plates of the same medium in which they were enriched, and following multiple rounds of subsequent re-streaking, over 100 bacterial isolates producing morphologically distinct colonies were obtained. Axenic cultures of all isolates were confirmed by microscopic observation and colony uniformity, and mid-logarithmic phase cultures of each were preserved at −80°C in medium containing 10% (v/v) dimethyl sulfoxide (DMSO). To focus the study, two isolates (strains 12SL-E129 and SL14, described below) that grew especially well and produced morphologically distinct colonies were selected for detailed physiological characterization, partial 16S rRNA sequencing, and phylogenetic analyses.

### 2.2 Microscopy

Cell morphology and arrangement were determined using a Leica Microsystems model DM1000 phase-contrast microscope, as previously described (Hayward et al., 2021). Motility in isolated strains was determined by microscopic observation and visual assessment of stab inoculations in semi-solid (0.35% agar) sulfide-indole-motility (SIM) medium (Becton, Dickinson and Company, Sparks, MD, USA; Zimbro et al., 2009).

### 2.3 Physiological studies

Growth temperature responses for isolated strains were determined spectrophotometrically by measuring the OD_540_ of triplicate cultures over temperatures ranging from 0–45°C. Absorbance measurements were taken immediately after inoculation of liquid tube cultures of modified medium R2A containing 2% NaCl and again after 7 days of incubation. The differences between the initial and final absorbances of the triplicate tubes at each temperature were averaged and compared to determine the optimum growth temperature for each isolate. The temperature supporting the highest cell density for each isolate was assigned a value of 100, and the percentage of maximum growth was calculated for each remaining temperature.

To determine salinity tolerances and optima, triplicate cultures of each isolate were established in modified medium R2A supplemented with final concentrations of NaCl ranging from 0 to 10% (w/v) in increments of 1%. Cultures were incubated at 20°C for either 5 days (strain SL14) or 7 days (strain 12SL-E129), and growth was evaluated as OD_540_ versus a time zero (T_0_) measurement. The NaCl concentration supporting the highest cell density for each isolate was assigned a value of 100, and the percentage of maximum growth was calculated for each remaining salinity value.

To determine the pH tolerance and optimum of each isolate, liquid cultures of modified medium R2A containing 2% NaCl and ranging in pH from 5 to 12 were established in triplicate. Buffers for each tested pH were added at a final concentration of initially 10 mM, but this was later adjusted to 25 mM to increase pH stability in the growing cultures. Buffers were used according to Asao et al. (2011) and included MES (pH 5–6), MOPS (pH 6.5–7.5), Bicine (pH 8–8.5), CAPSO (pH 9–10), and CAPS (pH 11–12). OD_540_ measurements were taken at T_0_ and again after seven (strain 12SL-E128) or three (strain SL14) days of growth. The differences between the initial and final absorbances were averaged for each pH value to determine the optimum pH for each isolate. The pH with the highest cell density was assigned a value of 100, and the percentage of maximum growth was calculated for each remaining pH value to define the pH range for each isolate.

Because of its phylogenetic affiliation with phototrophic members of the *Paracoccaceae*, strain 12SL-E129 was tested for growth under phototrophic conditions (anoxic with 50 μE·m^−2^·s^−1^ incandescent light) in 17-ml screw-capped tubes completely filled with medium RV5 (Milford et al., 2000) and incubated at 25°C. Controls in medium RV5 incubated aerobically both in the dark and in the light were also established. In addition, aerobic carbon source utilization was investigated for strain 12SL-E129 and compared to the carbon source profile of close relatives of the genus *Roseinatronobacter*. Usable carbon sources were determined in a mineral salts medium supplemented with sterile stock solutions of individual carbon sources (3–10 mM, final concentration), as previously described (Vander Schaaf et al., 2015).

Because of its phylogenetic affiliation with pathogenic members of the genus *Vibrio*, strain SL14 was inoculated onto sheep blood agar (SBA) plates and incubated at 20°C to assay for hemolytic activity. Cultures were visually inspected after cells reached stationary phase (48–72 h) to determine hemolysis.

### 2.4 Molecular methods

Community diversity analyses of *Bacteria* and *Archaea* inhabiting Soap Lake mixolimnion (3 m) and monimolimnion (23 m) waters using high-throughput DNA sequencing technology were performed by Charles River Laboratories (Newark, Delaware, USA) by first concentrating either 15 mL (3 m water) or 10 mL (23 m water) of the sample with an Amicon® Ultra-15 centrifuge filter, and total DNA was extracted with a Qiagen extraction kit. Library preparation for each sample was performed using an Illumina 16S (V1–V3) targeted amplicon DNA prep kit with universal primers 5F: TGGAGAGTTTGATCCTGGCTCAG and 527R: GTATTACCGCGGCTGCTGGCAC. Next generation sequencing was conducted using MiSeq (300 × paired end reads) Illumina chemistries. FastQ files were uploaded to the One Codex cloud-based bioinformatics pipeline,^1^ which compares against the National Center for Biotechnological Information (NCBI) RefSeq database. Totals of 2,413,928 (3 m water sample) and 2,681,128 (23 m water sample) reads were generated. Alpha (within sample) diversity metrics were calculated for each depth as Shannon and Simpson diversity indexes using the One Codex platform. Similarly, beta (between samples) diversity metrics were calculated both as Bray–Curtis dissimilarity and mean Aitchison distance using the One Codex platform.

Phylogenetic determinations for isolated strains were made by employing either FulSeq® (strain 12SL-E129) or BacSeq® (strain SL14) polymerase chain reaction (PCR) amplification of small-subunit (16S) ribosomal RNA genes, as previously described (Pop et al., 2025), which were then sequenced using the Sanger dideoxy method and identified using the Accugenix® library (Charles River Laboratories, Newark, DE). Initial taxonomic determinations were then confirmed using the nucleotide Basic Local Alignment Search Tool (BLAST) function of the NCBI database (Johnson et al., 2008).

Further phylogenetic analyses were performed using MEGA version 11 (Tamura et al., 2021). Type strains closely related to Soap Lake isolates were identified by cross-referencing BLAST 16S rRNA gene sequence searches with the List of Prokaryotic Names with Standing in Nomenclature (LPSN) website (https://bacterio.net; Parte, 2018). Neighbor-joining phylogenetic trees were generated using MEGA11, as described in their corresponding figure legends.

DNA sequences from 16S rRNA amplicon analyses performed for this study were deposited into the NCBI^2^ sequence read archive (SRA) database as BioProject accession PRJNA1279256. High-throughput (16S rRNA) data obtained for the 3-m and 23-m Soap Lake water samples were accessioned as SRX29246298 and SRX29246299, respectively. The partial 16S rRNA gene sequences for strains 12SL-E129 and SL14 were deposited into GenBank (NCBI) with accession numbers PV362398 and PV382118, respectively.

## 3 Results

### 3.1 Community diversity analysis

**Figure 2:**
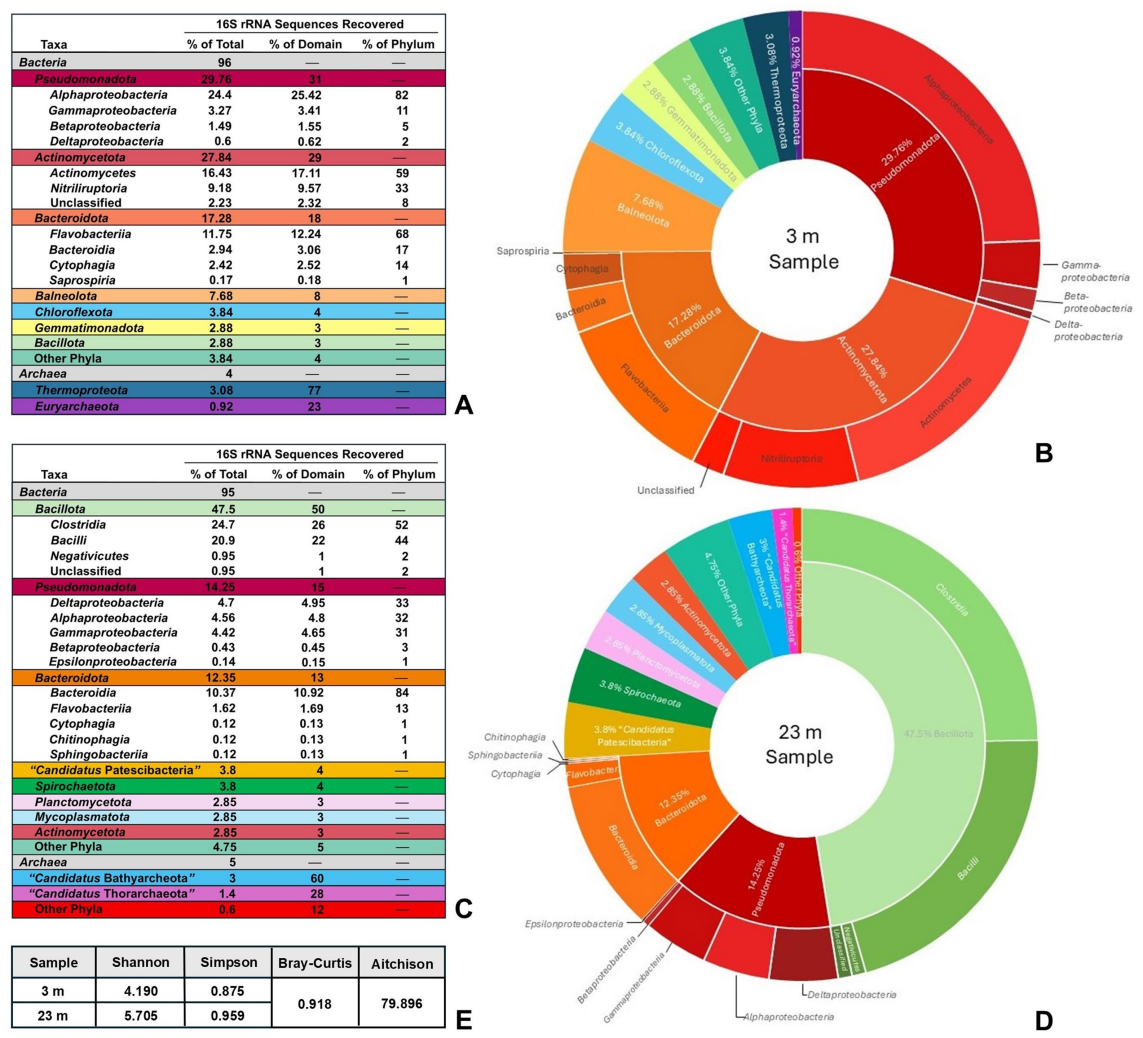
Soap Lake community sampling analysis. **(A)** Total taxonomic diversity of recovered DNA sequences from a 3-m Soap Lake water sample. “Other Phyla” are those bacterial taxa that individually represented <1% of total sequences and includes *Acidobacteriota*, *Spirochaetota*, *Cyanobacteriota*, *Deinococcota*, and “*Candidatus* Kaiseribacteriota.” Identified classes of *Bacteria* are shown in uncolored rows under each corresponding phylum. **(B)** Graphical representation of data in **(A)** with inner ring showing phyla and outer ring showing classes (classes not shown for phyla representing <8% of total recovered sequences). **(C)** Total taxonomic diversity of recovered DNA sequences from a 23-m Soap Lake water sample. “Other Phyla” are those bacterial or archaeal taxa that individually represented <1% of total sequences and includes *Acidobacteriota*, *Balneolota*, *Chloroflexota*, *Nitrospirota*, *Fusobacteriota*, *Cyanobacteriota*, *Deinococcota*, and *Thermotogota*, as well as the DPANN archaeal superphylum. Identified classes of *Bacteria* are shown in uncolored rows under each corresponding phylum. **(D)** Graphical representation of data in **(C)** with inner ring showing phyla and outer ring showing classes (classes not shown for phyla representing <5% of total recovered sequences). **(E)** Alpha diversity (Shannon and Simpson indexes) and beta diversity (Bray–Curtis dissimilarity and mean Aitchison distance) metrics at species level, indicating a greater degree of prokaryotic diversity at 23 m than 3 m in the Soap Lake water column, as well as substantial differences in the diversity between the two depths.

Marked differences were apparent in the microbial taxa identified by high-throughput 16S rRNA gene sequencing of the highly oxic mixolimnion (3 m) waters compared with the deep, anoxic monimolimnion (23 m) waters. Microbial communities in the 3-m water sample showed greater species evenness but overall lower species diversity than the 23-m water sample. The microbial composition of the 3-m water was dominated by members of the phyla *Pseudomonadota* and *Actinomycetota*, which comprised 29.76 and 27.84%, respectively, of the total identified taxa (Figures 2A,B). By far, *Alphaproteobacteria* represented the largest portion (82%) of sequences from the phylum *Pseudomonadota*, followed by *Gammaproteobacteria* (11%), *Betaproteobacteria* (5%), and *Deltaproteobacteria* (2%); *Epsilonproteobacteria* were not identified in the 3-m sample (Figure 2A). Classes representing the greatest portion of the phylum *Actinomycetota* included *Actinomycetes* (59%) and *Nitriliruptoria* (33%) (Figure 2A).

Members of the phylum *Bacteroidota* also represented a substantial portion of the 3-m microbial community at 17.28% of total identified taxa, with members of the class *Flavobacteriia* representing the greatest portion (68%) of this phylum (Figure 2A). No other phylum of *Bacteria* comprised more than 8% of total bacterial diversity in the 3-m sample (Figures 2A,B). *Archaea* were represented by the phyla *Thermoproteota* and *Euryarchaeota*, which comprised only minor portions (3.08 and 0.92%, respectively) of the total detected diversity in the 3-m sample (Figures 2A,B). However, because the 16S rRNA primers used in the analysis were not optimized for *Archaea*, the percentages of total archaeal diversity may be underestimates, and this caveat also applies for archaeal sequences detected in the 23-m Soap Lake water sample.

The 23-m water sample was dominated by species of the phylum *Bacillota*, which comprised nearly 50% of total diversity and consisted of nearly equal numbers of *Clostridia* and *Bacilli* (Figures 2C,D). Alpha (Shannon and Simpson diversity indexes) and beta (Bray–Curtis dissimilarity and mean Aitchison distance) diversity analyses showed that the 23-m water sample contained overall higher prokaryotic diversity than the 3-m sample, and species identified in the two samples differed substantially (Figure 2E). Although members of the *Pseudomonadota* and *Bacteroidota* were still well represented in the 23-m water at 14.25 and 12.35% of total diversity, respectively (Figures 2C,D), unlike in the 3-m sample, *Alphaproteobacteria*, *Deltaproteobacteria*, and *Gammaproteobacteria* comprised nearly equal portions of the *Pseudomonadota*, and *Epsilonproteobacteria* were detected (Figures 2C,D). Surprisingly, whereas members of the *Actinomycetota* comprised a major portion of taxa in the 3-m sample, this phylum represented just 2.85% of the total diversity in the 23-m water sample (Figure 2C). Archaeal phyla were also detected in the deep waters but differed from those identified in the 3-m sample. Most of the archaeal sequences in the 23-m water aligned with poorly characterized archaeal phyla, including “*Candidatus* Bathyarchaeota” and “*Candidatus* Thorarchaeota” (Figures 2C,D). The only identified archaeal phylum represented by sequences at both depths was *Euryarchaeota*.

Consistent with both the astonishingly high concentrations of sulfide in the monimolimnion of Soap Lake and its large percentage of *Clostridia* and *Deltaproteobacteria* relative to total diversity, sequences identified in the 23-m sample revealed a remarkable assemblage of both Gram-positive (*Desulfosporosinus*, *Desulfotomaculum*, *Desulfallas*, *Desulfohalotomaculum*, and *Desulfonispora*) and Gram-negative (*Desulfovibrio*, *Desulfobulbus*, *Desulfonatronovibrio*, *Desulfonatronobacter*, *Desulfuromusa*, *Desulfonatronum*, *Desulfococcus*, *Desulfosalsimonas*, and *Desulfobacter*) genera of sulfate-reducing bacteria (SRB). Sequences for SRB were not detected in the 3-m Soap Lake sample, presumably because of the sensitivity of SRB to O_2_. Sequences aligning with sulfur-oxidizing bacteria (SOB), including species of *Thiomicrospira* and *Thioalkalivibrio*, were also recovered from the 23-m sample.

Also absent from the 3-m sample but present in the 23-m sample where sulfide is available as a photosynthetic electron donor were phylotypes representing a substantial diversity of phototrophic purple nonsulfur bacteria, including *Rhodospirillum rubrum*, *Rhodobacter capsulatus*, *Rhodobacter veldkampii*, and *Rhodococcus jostii*. Also present were sequences identified as *Roseinatronobacter monicus* and *Roseinatronobacter bogoriensis*, two species that were most similar to our Soap Lake isolate, strain 12SL-E129 (discussed below), in phylogenetic analyses. Finally, in addition to phototrophic bacteria, a phylotype matching *Vibrio metschnikovii*, a species that was identified as the closest relative of our Soap Lake isolate, strain SL14 (also discussed below), was identified in the 23-m sample.

### 3.2 Isolation and morphology of Soap Lake isolates

Aerobic enrichment cultures established in 125-mL Erlenmeyer flasks containing a modified R2A medium (see Materials and methods) and adjusted to pH 9.5 yielded pure cultures of over 100 bacterial isolates through repeated streaking for isolation on agar plates of the same growth medium. Whereas most of these isolates remain uncharacterized, two were chosen for more detailed study based on their rapid growth, phenotypic properties, and phylogenetic distinctions: Strain 12SL-E129, a species of the genus *Roseinatronobacter* most closely related to *R. bogoriensis* and *R. monicus* based on 16S rRNA phylogeny; and strain SL14, a species of *Vibrio* phylogenetically related to *V. metschnikovii*, *V. aestuarianus*, and *V. salilacus*.

**Figure 3:**
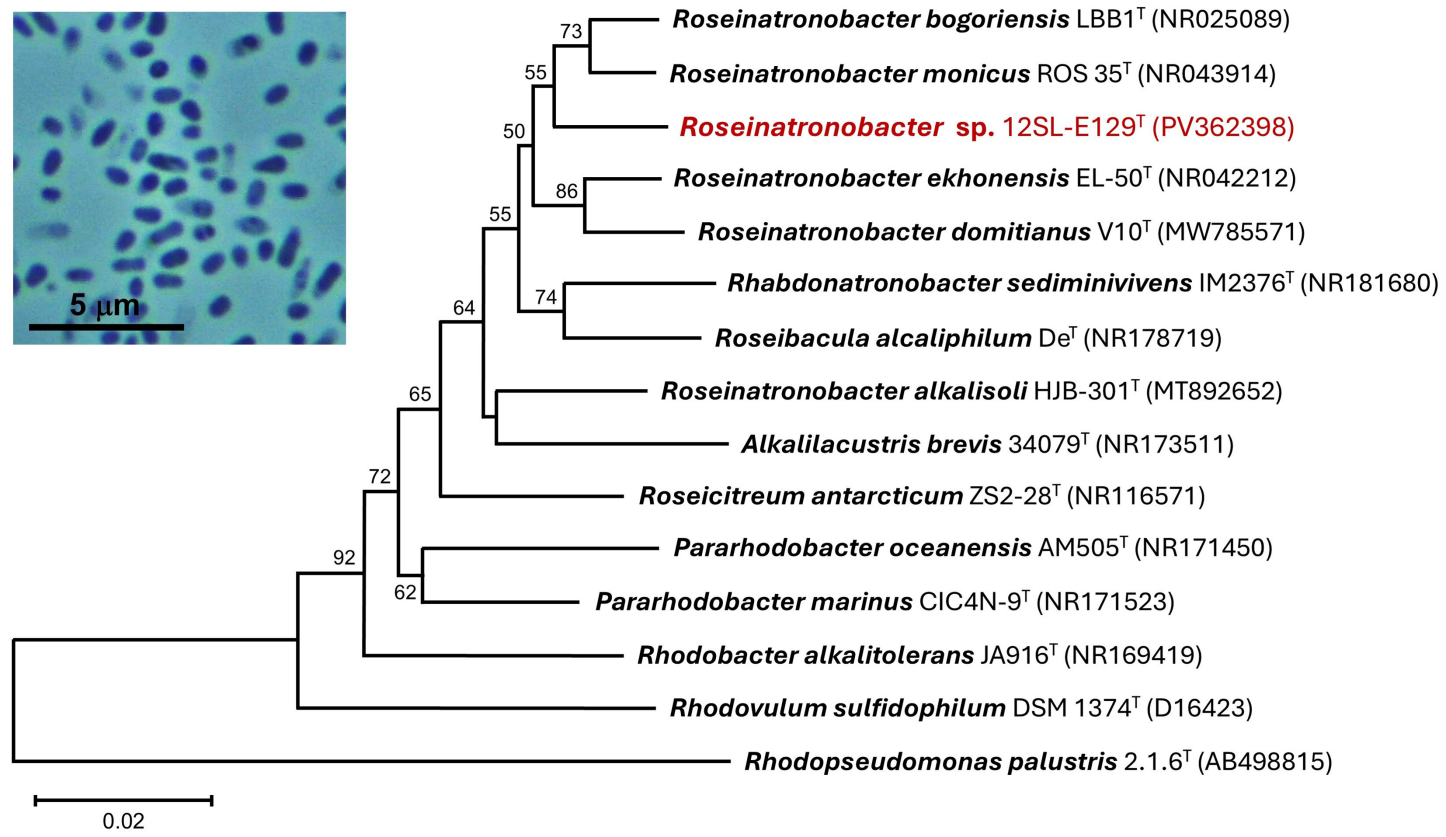
16S rRNA phylogenetic analysis of strain 12SL-E129 (red font). The sequenced 16S rRNA gene was aligned with closely related type strains using Muscle. The tree was constructed from 1,402 nucleotide positions in MEGA11 using the neighbor-joining method and the Kimura 2-parameter model. Bootstrap values (1,000 replicates) of >50% are indicated at the branching points, and GenBank accession numbers of all included taxa are shown in parentheses. The tree scale bar indicates a distance equivalent of 2 nucleotide changes per 100 nucleotides. *Inset photo*: Phase-contrast photomicrograph (1,000 × total magnification) of cells of strain 12SL-E129.

Strain 12SL-E129 grew well aerobically at room temperature (~22°C) and produced small (1–3 mm in diameter), round, cream- to pale pink-colored colonies with entire margins. Aerobic liquid cultures were unpigmented. Cells of strain 12SL-E129 were short (0.5–0.7 × 1–1.5 μm), pleomorphic, nonmotile coccobacilli and showed dark inclusions that were possibly polyphosphate granules (Figure 3, photo inset), as were observed in cells of its close relative *Roseinatronobacter monicus* (Boldareva et al., 2007). Both Gram staining and KOH testing (Buck, 1982) indicated the cells were Gram-positive, but subsequent phylogenetic analysis revealed the strain belonged to the Gram-negative family *Paracoccaceae*, described in more detail below.

**Figure 4:**
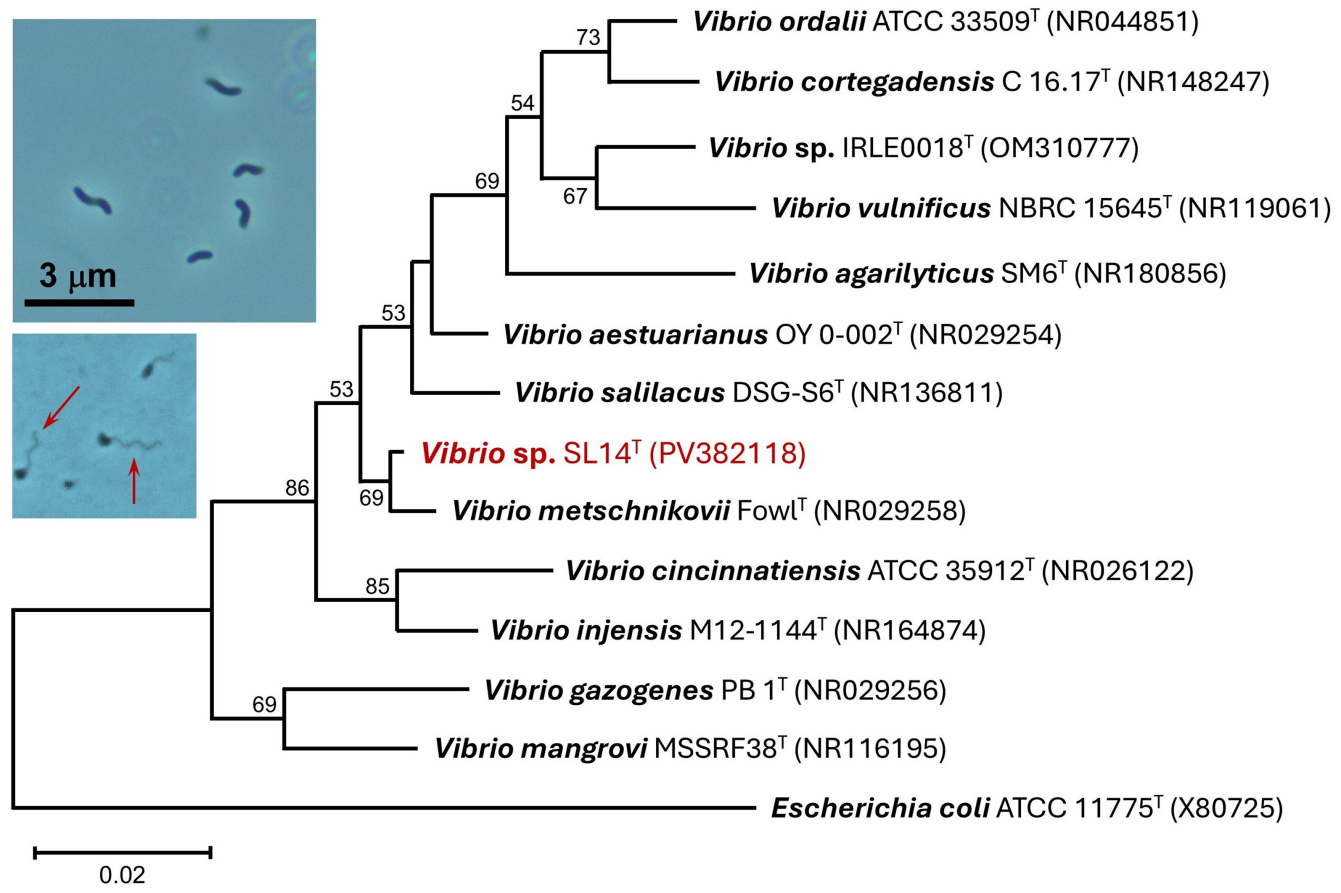
16S rRNA phylogenetic analysis of strain SL14 (red font). The sequenced 16S rRNA gene was aligned with closely related type strains using Muscle. The tree was constructed from 472 nucleotide positions in MEGA11 using the neighbor-joining method and the Kimura 2-parameter model. Bootstrap values (1,000 replicates) of >50% are indicated at the branching points, and GenBank accession numbers of all included taxa are shown in parentheses. The tree scale bar indicates a distance equivalent of 2 nucleotide changes per 100 nucleotides. *Inset photos*: Phase-contrast photomicrographs (1,000 × total magnification) of cells of strain SL14. Top photo: vibrio- to spirilla-shaped cells. Bottom photo: cells of strain SL14 with visible polar flagella (arrows).

Strain SL14 grew rapidly aerobically at room temperature in modified liquid R2A, with cultures producing visible turbidity overnight. Cells grown on plates of the same medium produced small- to medium-sized (2–5 mm in diameter), convex, cream-colored (unpigmented) colonies with undulate margins. Cells of strain SL14 were small (0.4–0.5 × 1–1.5 μm), vibrio- to spirilla-shaped, and highly motile from a single polar flagellum or possibly a tuft of bundled polar flagella (Figure 4, photo insets). When plated on sheep blood agar, colonies of strain SL14 were larger and darker in color and also showed extensive *β*-hemolysis, completely lysing the red blood cells in the medium surrounding colonies (data not shown).

### 3.3 Phylogeny of isolates

Analysis of the nearly complete 16S rRNA gene sequence of strain 12SL-E129 revealed it to group within the family *Paracoccaceae*. A neighbor-joining phylogenetic tree embedded strain 12SL-E129 firmly within a clade containing species of the genus *Roseinatronobacter*, with its highest sequence identities aligning with *Roseinatronobacter* (formerly *Rhodobaca*) *bogoriensis* (97.67%) and *Roseinatronobacter monicus* (97.27%) (Figure 3), both of which were isolated from other alkaline soda lakes (Milford et al., 2000; Boldareva et al., 2007). Interestingly, a second strain of *R. bogoriensis*, strain SLB, was isolated from Soap Lake water from a depth of 22 m (Asao et al., 2011). A BLAST analysis showed the 16S rRNA gene sequence of *R. bogoriensis* strain SLB to be 97.27% identical to that of strain 12SL-E129.

Microscopic observations showed that cells of strain SL14 exhibited the hallmark phenotypic properties of species of *Vibrio* (e.g., vibrio/spirilla cell morphology and vigorous flagellar swimming motility; Figure 4 photo inset), and indeed, a phylogenetic analysis confirmed its taxonomic status as belonging to this genus. Its closest cultured relatives were the halotolerant species *V. metschnikovii*, *V. salilacus*, and *V. aestuarianus*, which showed sequence identities ranging from 95.76–98.76% using partial 16S rRNA gene sequences (Figure 4).

### 3.4 Physiology of isolates

**Figure 5:**
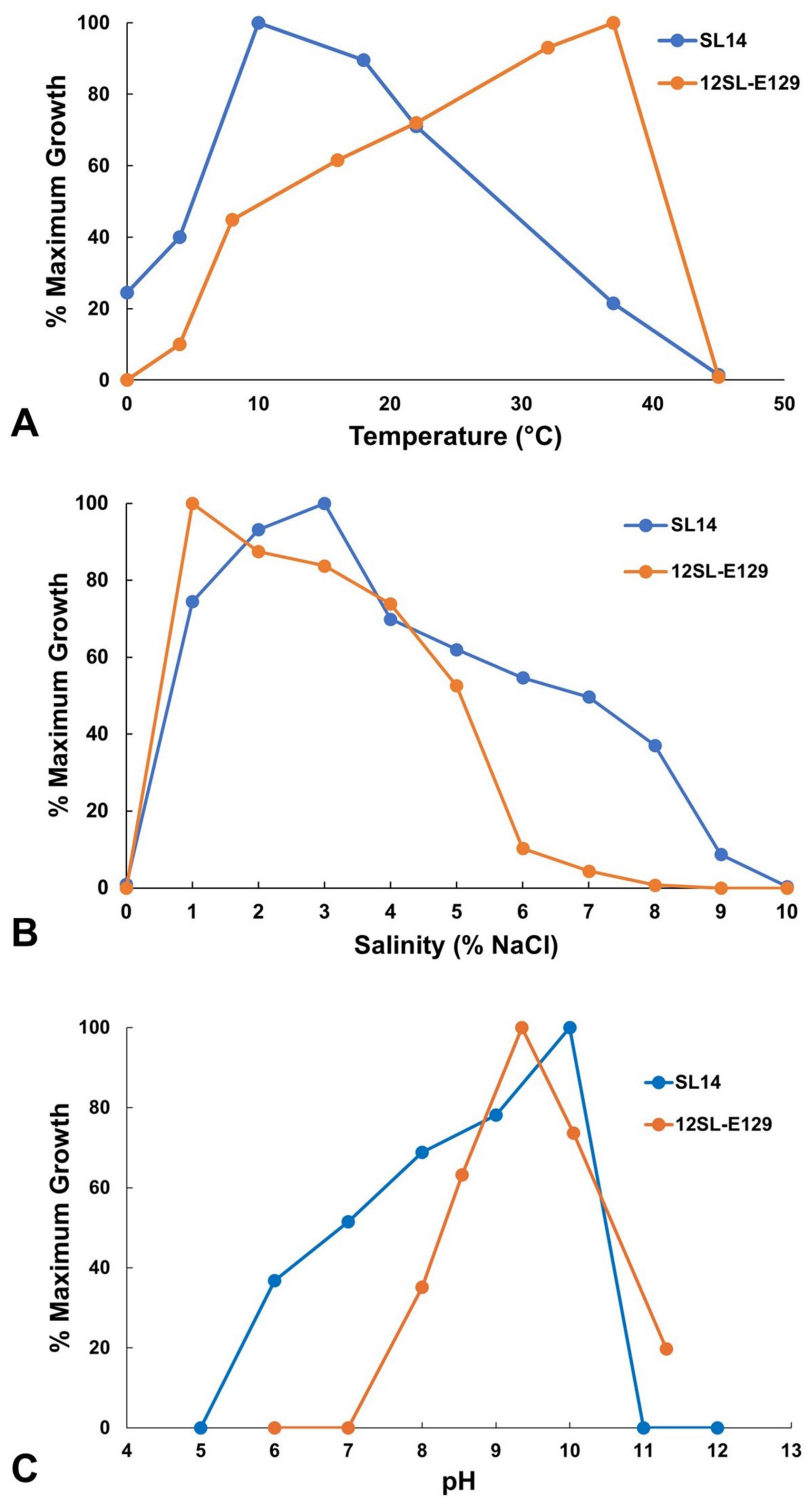
Growth of Soap Lake bacterial isolates as a function of **(A)** temperature, **(B)** salinity, and **(C)** pH. Growth is presented as a percent of the maximum absorbance (OD_540_) averaged from triplicate cultures for each parameter. Under optimal conditions, final turbidity maxima (OD_540_) reached 0.416 for strain 12SL-E129 and 0.722 for strain SL14, with mean generation times of 20 h and 5.5 h, respectively.

*Roseinatronobacter* strain 12SL-E129 exhibited a broad growth temperature range. Slow growth was evident at 4°C, with growth rates steadily increasing to optimal at 37°C; no growth of strain 12SL-E129 occurred at 0 or 45°C (Figure 5A). By contrast, the growth temperature profile of *Vibrio* strain SL14 differed substantially from that of strain 12SL-E129. Unexpectedly, strain SL14 exhibited a cold-adapted, psychrotolerant phenotype, with optimal growth occurring at 10°C (Figure 5A). Remarkably, slow to moderate growth of strain SL14 occurred at 0°C, with rates being slightly faster than those observed at 37°C, a temperature at which the organism was clearly stressed (Figure 5A).

Both of the Soap Lake isolates described in this study had an absolute requirement for NaCl, and therefore unlike their closest relatives (Figures 3, 4), they are classified as true halophiles. Although no growth of either isolate occurred in medium completely lacking NaCl, copious growth of both strains was observed with the addition of 1% (w/v) NaCl, and *Roseinatronobacter* strain 12SL-E129 grew best with this salt concentration (Figure 5B). *Vibrio* strain SL14 was more halophilic than strain 12SL-E129 (Figure 5B). Growth of strain SL14 was optimal with 3% NaCl, and whereas growth of strain 12SL-E129 was barely measurable at 7% NaCl, slow growth of strain SL14 could still be detected in medium containing up to 9% NaCl (Figure 5B).

The pH optima, and especially the pH ranges, also differed between strains 12SL-E129 and SL14. Although *Roseinatronobacter* strain 12SL-E129 had a lower pH optimum than *Vibrio* strain SL14 (pH 9 versus pH 10), it exhibited an overall more alkaliphilic phenotype, growing within a range of pH 8–11.5 but with no growth at pH 7 (Figure 5C). Compared to *R. bogoriensis* and *R. monicus*, strain 12SL-E129 exhibited a similar pH optimum but a higher pH maximum (Milford et al., 2000; Boldareva et al., 2007). In contrast to strain 12SL-E129, no growth of *Vibrio* strain SL14 was observed at pH 11 even though optimal growth occurred at pH 10 (Figure 5C). In addition, strain SL14 had an especially broad pH range, growing slowly even at pH 6 (Figure 5C), a characteristic shared with *V. salilacus* (Zhong et al., 2015).

Because of the phylogenetic grouping of *Roseinatronobacter* strain 12SL-E129 within a genus containing phototrophic purple nonsulfur bacteria, we investigated both usable carbon sources and the potential for pigment biosynthesis and phototrophic (anoxic/light) growth by this isolate. Strain 12SL-E129 was able to use a variety of carbon sources aerobically in darkness. The overall carbon utilization profile resembled that reported by Milford et al. (2000) for *R. bogoriensis*, with strain 12SL-E129 having a preference for sugars and fatty/organic acids but being unable to metabolize alcohols (Table 1). Growth of strain 12SL-E129 in medium RV5, which was used for most culture-based experiments on *R. bogoriensis* (Milford et al., 2000), was good under aerobic conditions, but no growth of strain 12SL-E129 occurred anaerobically under incandescent illumination in this medium. In addition, there was no evidence of any photosynthetic pigment production in cultures of strain 12SL-E129 under any growth conditions tested.

**Table 1:** Carbon source utilization profile of strain 12SL-E129 and close relatives of the genus *Roseinatronobacter* under aerobic dark conditions.^a^

Carbon Source	Strain 12SL-E129^b^	*R. bogoriensis* ^c^	*R. monicus* ^d^	*R. domitianus* ^e^	*R. ekhonensis* ^f^
Sugars/sugar alcohols					
Glucose	(+)	+	−	ND	+
Fructose	(+)	+	+	+	−
Galactose	−	ND	+	ND	ND
Lactose	−	−	+	ND	+
Sucrose	+	+	+	ND	ND
Ribose	−	−	ND	ND	ND
Mannose	(+)	ND	ND	ND	ND
Maltose	(+)	ND	+	−	+
Cellobiose	+	ND	ND	ND	ND
Melibiose	−	ND	ND	+	−
Arabinose	+	ND	+	ND	ND
Raffinose	(+)	ND	ND	ND	ND
Xylose	(+)	+	ND	ND	ND
Mannitol	(+)	+	+	ND	ND
Adonitol	−	ND	ND	ND	ND
Fatty/organic acids					
Acetate	(+)	(+)	+	ND	ND
Butyrate	+	+	−	+	−
Propionate	(+)	+	+	ND	ND
Pyruvate	(+)	+	+	ND	ND
Lactate	(+)	(+)	+	+	−
Fumarate	(+)	+	ND	ND	ND
Malate	+	+	+	ND	ND
Citrate	(+)	−	+	−	+
Formate	−	−	ND	ND	ND
Succinate	−	+	−	ND	−
Benzoate	−	−	−	ND	ND

a Alcohols (ethanol, methanol, butanol, and propanol) were not utilized by strain 12SL-E129 *R. bogoriensis* and *R. monicus* and were not tested in nutritional experiments with the other two species.

b Aerobic growth of strain 12SL-E129 was assessed as the difference of the time zero absorbance (OD_540_) from the final absorbance using the following scale: ++, ∆ OD_540_ > 0.15; +, ∆ OD_540_ 0.051–0.150; (+), ∆ OD_540_ 0.021–0.050; −, ∆ OD_540_ 0–0.02. Carbon sources had the following final concentrations: benzoate, lactose, 3 mM; maltose, sucrose, fructose, glucose, galactose, butyrate, succinate, 5 mM; all others, 10 mM. ND, not determined.

c Data from Milford et al. (2000).

d Data from Boldareva et al. (2007).

e Data from Gattoni et al. (2023).

f Data from Labrenz et al. (2009).

## 4 Discussion

Diversity analyses of the two Soap Lake water samples surveyed in this study revealed an overall greater degree of prokaryotic diversity in the deep (23-m) monimolimnion water than in the shallower (3-m) mixolimnion sample. A similar observation was noted in an earlier study by Dimitriu et al. (2008), in which denaturing gradient gel electrophoresis of 16S rRNA genes showed an increase of Soap Lake prokaryotic diversity with depth. In addition, both the present study and that conducted by Dimitriu et al. (2008) showed substantial differences in the composition of prokaryotic taxa identified in warmer, oxic Soap Lake waters compared to colder, anoxic, and highly saline 23-m lake water (note that whereas a 3-m mixolimnion sample was analyzed in this study, the 2008 study used a 5-m sample; both studies used a monimolimnion sample from a depth of 23 m). The observed distinctions in microbial community composition between the shallower and deeper waters are presumably linked to the dramatic physicochemical differences between the mixolimnion and monimolimnion of Soap Lake.

In the study by Dimitriu et al. (2008), a 16S rRNA gene clone library constructed from late-July (2003) samples revealed a predominance of *Alpha*- and *Gammaproteobacteria* in 5-m mixolimnion water and a predominance of *Bacillota* (i.e., “low G + C Gram-positive bacteria”) and *Bacteroidota* (i.e., “CFB”; *Cytophaga-Flexibacter-Bacteroides*) in 23-m monimolimnion water. These results are similar, but not identical, to those obtained in the present study on Soap Lake samples also collected in late July but nearly 20 years later. Both studies showed a predominance of *Proteobacteria* in the mixolimnion and the disappearance of *Actinobacteria* and *Chloroflexota* with depth, as well as an absence of *Epsilonproteobacteria* in the mixolimnion but detection of these bacteria in 23-m water. However, whereas Dimitriu et al. (2008) detected good representation (>10%) of “low G + C Gram-positive bacteria” in 5-m mixolimnion water, *Bacillota* represented only a small portion (<3%) of sequences identified in 3-m mixolimnion water in the present study. Considering that both studies revealed an increase of *Bacillota* with depth, this variation may simply be a reflection of the 2-m difference in depth of the two samples analyzed.

In both the work of Dimitriu et al. (2008) and in this study, sequences of *Alphaproteobacteria* diminished dramatically with depth, but *Gammaproteobacteria* remained a significant portion of detected sequences in the monimolimnion at approximately 5% of the total. Among the sequences of *Gammaproteobacteria* detected in our 23-m sample were sulfur-oxidizing chemolithotrophic bacteria (SOB), including species of *Thiomicrospira* and *Thioalkalivibrio*. This finding was consistent with observations by Sorokin et al. (2007) in which SOB, particularly species of *Thioalkalivibrio*, were both detected and cultured from both chemocline and monimolimnion Soap Lake waters. In contrast to results from Dimitriu et al. (2008), representation of *Deltaproteobacteria* in our samples increased notably with depth from <1% of total sequences at 3 m to nearly 5% at 23 m. As previously discussed, this observation is presumably due in large part to the presence of an abundance of remarkably diverse sulfate-reducing bacteria that include both Gram-negative (*Deltaproteobacteria*) and Gram-positive (*Clostridia*) taxa. As SRB are generally strict anaerobes, predictably, none of these sequences was detected in the oxic, 3-m Soap Lake sample. Considering the presence of both dissolved O_2_ and sulfide in the chemocline and the abundance of available sulfate and dissolved sulfide in the monimolimnion, it is likely that microbial sulfur cycling drives a significant portion of the metabolic processes in the deeper waters of Soap Lake. Previous studies describing the culture of both SOB (Sorokin et al., 2007) and SRB (Dimitriu et al., 2008) from Soap Lake support this idea, and we also have readily enriched both lactate-oxidizing SRB and thiosulfate-oxidizing bacteria from Soap Lake water samples from these depths (unpublished results).

The detection of purple anoxygenic phototrophs in Soap Lake deep water was consistent with previous studies in which alkaliphilic purple sulfur bacteria, including members of the families *Thiocapsaceae*, *Chromatiaceae*, and *Ectothiorhodospiraceae* (all of the order *Chromatiales*), were cultured from a 22-m Soap Lake water sample (Asao et al., 2007, 2011). *Roseinatronobacter* strain 12SL-E129 was isolated from a Soap Lake water sample taken from a depth of 12 m, and its closest phylogenetic relatives, *Roseinatronobacter bogoriensis* and *Roseinatronobacter monicus*, were also isolated from alkaline soda lakes. The type strain of *R. bogoriensis* was isolated from a water/sediment sample from Lake Bogoria in the African Rift Valley (Milford et al., 2000), and *R. monicus* was obtained from the water column of Mono Lake in California, USA (Boldareva et al., 2007). Interestingly, as mentioned above, a second strain of *R. bogoriensis* (strain SLB) was isolated from a 22-m Soap Lake water sample by Asao et al. (2011), and therefore our detection of this species in Soap Lake water from a depth of 23 m was not unexpected.

*R. bogoriensis* and *R. monicus* both synthesize bacteriochlorophyll (BChl) *a* as a major pigment. *R. bogoriensis* can grow aerobically on a variety of organic carbon sources, but it is also capable of phototrophic growth (Milford et al., 2000). However, phototrophic growth is not accompanied by autotrophic CO_2_ assimilation in *R. bogoriensis*, as the organism lacks genes encoding autotrophic pathways (Madigan et al., 2023). *R. monicus* is also obligately heterotrophic, but unlike *R. bogoriensis*, *R. monicus* is incapable of anaerobic phototrophic growth despite its production of BChl *a* (Boldareva et al., 2007). Therefore, similar to *R. monicus* (Boldareva et al., 2007), *Roseinatronobacter ekhonensis* (Labrenz et al., 2009), *Roseinatronobacter thiooxidans* (Sorokin et al., 2000), and *Roseinatronobacter alkalisoli* (Hu et al., 2024), strain 12SL-E129 appears to be an obligately aerobic heterotroph incapable of phototrophic growth. This is in contrast to other members of the genus *Roseinatronobacter*, including *R. domitianus* (Gattoni et al., 2023) and all strains of *R. bogoriensis* (Milford et al., 2000; Boldareva et al., 2008; Asao et al., 2011; Madigan et al., 2023), which are all capable of photoheterotrophic growth.

The growth temperature optimum for strain 12SL-E129 (37°C) was similar to that of the type strain of *R. bogoriensis* (39°C); however, the growth temperature range was much broader for strain 12SL-E129. Growth of *R. bogoriensis* occurred over an unusually narrow temperature range of just 30–43°C (Milford et al., 2000), a phenotype that may be a product of the year-round, consistently mild-to-warm equatorial temperatures experienced by its Lake Bogoria (Kenya) habitat. By contrast, strain 12SL-E129—isolated from a depth of 12 m where temperatures typically range from 12 to 20°C—exhibited moderate growth at 10°C and grew slowly at 4°C (Figure 5A), observations that may be indicative of the versatility required for the more seasonal climate experienced by Soap Lake. Growth versus temperature data were not provided for Soap Lake isolate *R. bogoriensis* strain SLB (Asao et al., 2011).

Growth of *Roseinatronobacter* strain 12SL-E129 in cultures required the addition of NaCl, an observation that differed from the physiology of *R. bogoriensis* and *R. monicus*, both of which were capable of growing in NaCl-free media (Milford et al., 2000; Boldareva et al., 2007). Despite its requirement for Na^+^, strain 12SL-E129 was unable to grow in medium containing more than 7% (w/v) NaCl (Figure 5B), a salinity maximum slightly lower than the 10 and 8% salinity maxima recorded for *R. bogoriensis* and *R. monicus*, respectively (Milford et al., 2000; Boldareva et al., 2007). Thus, although its growth in cultures depended on the presence of NaCl, strain 12SL-E129 displayed a rather narrow salinity range for growth.

The closest cultured relatives of *Vibrio* strain SL14 based on 16S rRNA phylogeny were *V. metschnikovii*, *V. salilacus*, and *V. aestuarianus* (Figure 4). *V. salilacus* was isolated from haloalkaline Dasugan Lake (3.1% salinity, pH 9.2) in the arid hills of north central China (Zhong et al., 2015), and therefore of the three species, shares the greatest ecological similarity to strain SL14. *V. metschnikovii* has a long history (Gamaleia, 1888) and was recently classified as an emerging human pathogen (Bartlett et al., 2022; Huang et al., 2023). *V. aestuarianus* is a marine species that has been associated with bivalve, especially oyster, mortality (Garcia et al., 2021). As at least some of its closest relatives are known to be pathogenic in humans or other animal species (some strains of *V. metschnikovii* are categorized as biosafety level 2), strain SL14 may possess some degree of virulence, a possibility underscored by clear *β*-hemolytic activity when the strain was grown on sheep blood agar.

*Vibrio* strain SL14 exhibited a temperature optimum of 10°C but was still capable of vigorous growth at 18°C (Figure 5A). The strain was isolated from a depth of 14 m, where Soap Lake water temperatures range from about 10–20°C, depending on the season (Edmondson and Anderson, 1965). Species of *Vibrio* often do exhibit a psychrotolerant phenotype (both *V. aestuarianus* and *V. salilacus*, but not *V. metschnikovii*, are capable of growth at 4°C; Zhong et al., 2015), and therefore strain SL14 appears to be well adapted to its *in situ* Soap Lake temperatures.

Strain SL14 showed a greater degree of adaptation to high salinity than strain 12SL-E129 and was able to grow in medium containing up to 9% NaCl. This is also a greater degree of halotolerance than that exhibited by its closest relatives *V. metschnikovii*, *V. aestuarianus*, and *V. salilacus*, none of which are able to grow in medium containing ≥8% NaCl (Zhong et al., 2015). Nevertheless, for a true halophile with a growth requirement for NaCl, strain SL14, like strain 12SL-E129, was unexpectedly sensitive to moderate salinity levels (Figure 5B). This characteristic of exhibiting a halophilic phenotype but with tolerance for only modest concentrations of NaCl (<10% in the case of these strains; Figure 5B) may be a reflection of the unusual stability of the Soap Lake meromictic water column, in which salinity is constant but remains relatively low (≤20 g L^−1^; Sorokin et al., 2007) above the chemocline where these strains originated.

## 5 Conclusion

It is generally understood that biological productivity and diversity are substantially more limited in extreme environments than in ecosystems that maintain more hospitable physicochemical conditions. However, haloalkaline aquatic environments may be an exception in this regard, as many of these ecosystems are highly productive (Asao et al., 2011). In addition, the impressive scope of the microbial biodiversity inhabiting these ecosystems is becoming fully realized through the broader application of molecular-based microbial community analyses. The results of this study highlight Soap Lake as a rich source of a broad diversity of microorganisms able to carry out biochemical transformations under highly saline, alkaline conditions, potentially also at reduced temperatures. Although the number of studies highlighting the microbiology of alkaline lakes has expanded over the past 25 years, intensifying research efforts in this area should prove fruitful. In addition to continued characterization of the numerous strains already isolated in this work, future research efforts will benefit by employing targeted aerobic and anaerobic enrichment strategies informed by the phylogenomic data presented here. Building upon the growing range of alkaliphilic microorganisms having intriguing biotechnological utility and potential (Sarethy et al., 2011; Mamo and Mattiasson, 2020; Rawat et al., 2024; Sarwa et al., 2024; Kour et al., 2025), future investigations of Soap Lake microbiota will almost certainly yield additional novel microorganisms able to perform valuable tasks having commercial, industrial, or environmental applications.

## Data Availability

The datasets generated for this study can be found in the NCBI GenBank Nucleotide Repository (https://www.ncbi.nlm.nih.gov/nucleotide/). The accession numbers and strain names are presented in Figures 3, 4.

## Footnotes

^1^ www.onecodex.com

^2^ https://www.ncbi.nlm.nih.gov/
